# Supplementary material for: A cellular and molecular atlas reveals the basis of chytrid development
Source: eLife. 2022 Mar 1;11:e73933. doi: 10.7554/eLife.73933 (PMC8887899; doi:10.7554/eLife.73933)
Supplement: Supplementary file 1. [file elife-73933-supp1.docx]

**Supplementary Table 1.** Volumetric quantities of cellular structures recorded across chytrid life stages. Data given to 3 decimal places.

| **Cellular**  **Structure** | **Chytrid Life stage – Volume in µm^3^** | | | | | | | | | |
| --- | --- | --- | --- | --- | --- | --- | --- | --- | --- | --- |
|  | **Zoospore**  **(*n* = 5)** | **±**  **S.D** | **Germling**  **(*n* = 5)** | **±**  **S.D** | **Immature**  **Thallus (*n* = 5)** | **±**  **S.D** | **Imm. Thall. Apophysis (*n* = 5)** | **±**  **S.D** | **Dev. Zoospore**  **(*n* = 5)** | **±**  **S.D** |
| **Total Volume** | 20.749 | 1.687 | 33.991 | 2.042 | 1116.291 | 206.198 | 12.179 | 5.951 | 21.455 | 0.590 |
| **Cell Wall** | 0.000 | 0.000 | 2.594 | 0.425 | 26.448 | 3.112 | 1.326 | 0.600 | 0.000 | 0.000 |
| **Cytosolic Lipid** | 0.894 | 0.558 | 1.889 | 1.127 | 3.754 | 1.983 | 0.115 | 0.183 | 1.451 | 0.183 |
| **Endomembrane** | 0.195 | 0.070 | 0.479 | 0.410 | 30.829 | 11.485 | 1.390 | 0.729 | 0.346 | 0.072 |
| **Glycogen** | 0.332 | 0.253 | 0.433 | 0.138 | 104.359 | 27.516 | 0.000 | 0.000 | 1.190 | 0.334 |
| **Golgi Apparatus** | 0.000 | 0.000 | 0.106 | 0.101 | 4.498 | 0.898 | 0.153 | 0.149 | 0.079 | 0.033 |
| **Microbodies** | 0.226 | 0.189 | 0.336 | 0.118 | 2.025 | 1.857 | 0.000 | 0.000 | 0.194 | 0.253 |
| **Mitochondria** | 1.937 | 0.170 | 3.092 | 0.347 | 78.194 | 14.392 | 0.977 | 0.934 | 1.803 | 0.113 |
| **Nucleus** | 2.143 | 0.372 | 4.129 | 0.293 | 63.155 | 30.682 | 0.000 | 0.000 | 1.544 | 0.057 |
| **Peripheral Bodies** | 0.000 | 0.000 | 0.580 | 0.125 | 3.620 | 0.753 | 0.121 | 0.270 | 0.000 | 0.000 |
| **Ribosome Cluster** | 4.228 | 0.522 | 0.000 | 0.000 | 0.000 | 0.000 | 0.000 | 0.000 | 0.000 | 0.000 |
| **Rumposome** | 0.053 | 0.005 | 0.024 | 0.014 | 0.000 | 0.000 | 0.000 | 0.000 | 0.028 | 0.003 |
| **Striated Inclusion** | 0.032 | 0.031 | 0.000 | 0.000 | 0.000 | 0.000 | 0.000 | 0.000 | 0.000 | 0.000 |
| **Vacuole-bound Lipid** | 0.000 | 0.000 | 0.000 | 0.000 | 42.568 | 20.506 | 0.132 | 0.090 | 0.000 | 0.000 |
| **Vacuoles excl. Lipid Contents** | 0.488 | 0.311 | 2.576 | 0.369 | 144.077 | 31.251 | 1.568 | 1.234 | 1.809 | 0.472 |
| **Vesicles** | 0.000 | 0.000 | 0.000 | 0.000 | 0.000 | 0.000 | 0.000 | 0.000 | 0.135 | 0.024 |
| **Total Assigned Organelles** | 10.527 | 1.399 | 16.240 | 0.607 | 503.526 | 89.771 | 5.781 | 2.508 | 8.579 | 0.632 |
| **Unassigned Cytosol** | 10.222 | 1.268 | 17.751 | 1.670 | 612.764 | 122.699 | 6.398 | 3.675 | 12.877 | 0.389 |
| **Vacuoles incl. Lipid Contents** | 0.488 | 0.341 | 2.576 | 0.369 | 186.645 | 40.006 | 1.700 | 1.240 | 1.809 | 0.472 |
| **Total Lipid Fraction *** | 0.909 | 0.558 | 1.889 | 1.127 | 46.322 | 21.419 | 0.247 | 0.189 | 1.451 | 0.183 |
| **Total Endomembrane Fraction **** | 0.275 | 0.375 | 4.018 | 0.802 | 227.616 | 51.755 | 3.363 | 1.212 | 2.563 | 0.445 |

****A functional category defined by the sum of cytosolic and vacuole-bound lipids.***

*****A functional category defined by the sum of the endomembrane, Golgi apparatus, microbodies, peripheral bodies, vacuoles incl. lipid contents, and vesicles.***
