# Supplementary material for: A cellular and molecular atlas reveals the basis of chytrid development
Source: eLife. 2022 Mar 1;11:e73933. doi: 10.7554/eLife.73933 (PMC8887899; doi:10.7554/eLife.73933)
Supplement: Supplementary file 2. [file elife-73933-supp2.docx]

**Supplementary Table 2.** Numerical quantities of cellular structures recorded across chytrid life stages. Data given to 3 decimal places.

| **Cellular**  **Structure** | **Chytrid Life stage – Volume in µm^3^** | | | | | | | | | |
| --- | --- | --- | --- | --- | --- | --- | --- | --- | --- | --- |
|  | **Zoospore**  **(*n* = 5)** | **±**  **S.D** | **Germling**  **(*n* = 5)** | **±**  **S.D** | **Immature**  **Thallus (*n* = 5)** | **±**  **S.D** | **Imm. Thall. Apophysis (*n* = 5)** | **±**  **S.D** | **Dev. Zoospore**  **(*n* = 5)** | **±**  **S.D** |
| **Total Volume** | NA | NA | NA | NA | NA | NA | NA | NA | NA | NA |
| **Cell Wall** | 0.000 | 0.000 | 1.000 | 0.000 | 1.000 | 0.000 | 1.000 | 0.000 | 0.000 | 0.000 |
| **Cytosolic Lipid** | 1.000 | 0.000 | 1.000 | 0.000 | 68.800 | 55.233 | 13.600 | 14.276 | 1.000 | 0.000 |
| **Endomembrane** | 57.000 | 14.782 | 88.800 | 18.336 | 1513.800 | 641.545 | 45.000 | 35.050 | 88.400 | 21.629 |
| **Glycogen** | 331.200 | 76.424 | 167.800 | 56.193 | 1075.000 | 137.099 | 0.000 | 0.000 | 91.400 | 32.601 |
| **Golgi Apparatus** | 0.000 | 0.000 | 1.400 | 1.342 | 62.200 | 13.180 | 1.800 | 1.095 | 1.400 | 0.548 |
| **Microbodies** | 1.400 | 0.548 | 1.400 | 0.548 | 10.800 | 11.692 | 0.000 | 0.000 | 1.200 | 0.447 |
| **Mitochondria** | 2.800 | 2.490 | 1.200 | 0.447 | 237.200 | 129.820 | 27.600 | 31.198 | 9.000 | 3.082 |
| **Nucleus** | 1.000 | 0.000 | 1.000 | 0.000 | 1.800 | 1.304 | 0.000 | 0.000 | 1.000 | 0.000 |
| **Peripheral Bodies** | 0.000 | 0.000 | 3.600 | 2.074 | 52.600 | 23.650 | 1.200 | 2.683 | 0.000 | 0.000 |
| **Ribosome Cluster** | 1.000 | 0.000 | 0.000 | 0.000 | 0.000 | 0.000 | 0.000 | 0.000 | 0.000 | 0.000 |
| **Rumposome** | 1.000 | 0.000 | 0.8 | 0.447 | 0.000 | 0.000 | 0.000 | 0.000 | 1.000 | 0.000 |
| **Striated Inclusion** | 0.600 | 0.548 | 0.000 | 0.000 | 0.000 | 0.000 | 0.000 | 0.000 | 0.000 | 0.000 |
| **Vacuole-bound Lipid** | 0.000 | 0.000 | 0.000 | 0.000 | 70.600 | 39.835 | 4.400 | 2.074 | 0.000 | 0.000 |
| **Vacuoles excl. Lipid Contents** | 8.000 | 1.581 | 4.200 | 3.271 | 69.800 | 37.164 | 19.800 | 14.856 | 12.200 | 9.497 |
| **Vesicles** | 0.000 | 0.000 | 0.000 | 0.000 | 0.000 | 0.000 | 0.000 | 0.000 | 53.600 | 8.905 |
| **Total Assigned Organelles** | 405.000 | 81.557 | 272.200 | 59.302 | 3163.600 | 756.752 | 114.400 | 62.408 | 260.200 | 48.561 |
| **Unassigned Cytosol** | NA | NA | NA | NA | NA | NA | NA | NA | NA | NA |
| **Vacuoles incl. Lipid Contents** | 8.000 | 1.581 | 4.200 | 3.271 | 69.800 | 37.164 | 19.800 | 14.856 | 12.200 | 9.497 |
| **Total Lipid Fraction *** | 1.000 | 0.000 | 1.000 | 0.000 | 139.400 | 60.789 | 18.000 | 15.922 | 1.000 | 0.000 |
| **Total Endomembrane Fraction **** | 75.400 | 19.424 | 99.400 | 19.501 | 1709.200 | 617.922 | 67.800 | 43.540 | 156.800 | 22.797 |

****A functional category defined by the sum of cytosolic and vacuole-bound lipids.***

*****A functional category defined by the sum of the endomembrane, Golgi apparatus, microbodies, peripheral bodies, vacuoles incl. lipid contents, and vesicles.***
