# Supplementary material for: A cellular and molecular atlas reveals the basis of chytrid development
Source: eLife. 2022 Mar 1;11:e73933. doi: 10.7554/eLife.73933 (PMC8887899; doi:10.7554/eLife.73933)
Supplement: Supplementary file 3. [file elife-73933-supp3.docx]

| **Cellular**  **Structure** | **Chytrid Life stage – Volumetric %** | | | | | | | | |
| --- | --- | --- | --- | --- | --- | --- | --- | --- | --- |
|  | **Zoospore**  **(*n* = 5)** | **±**  **S.D** | **Germling**  **(*n* = 5)** | **±**  **S.D** | **Immature**  **Thallus (*n* = 5)** | **±**  **S.D** | **Statistical Test used** | ***p*- Value** | **Posthoc Annotation** |
| **Total Volume** | 100.000 | 0 | 100.000 | 0 | 100.000 | 0.000 | NA | NA | NA |
| **Cell Wall** | 0.000 | 0.000 | 7.644 | 1.245 | 2.409 | 0.328 | Mann Whitney U | <0.01 | A B C |
| **Cytosolic Lipid** | 4.290 | 2.610 | 5.714 | 3.678 | 0.341 | 0.159 | Kruskal | <0.01 | A A B |
| **Endomembrane** | 0.948 | 0.353 | 1.371 | 1.112 | 2.691 | 0.597 | ANOVA | <0.01 | A A B |
| **Glycogen** | 1.590 | 1.213 | 1.265 | 0.376 | 9.399 | 1.969 | Kruskal | <0.01 | A A B |
| **Golgi Apparatus** | 0.000 | 0.000 | 0.321 | 0.313 | 0.414 | 0.104 | Mann Whitney U | >0.05 | A B B |
| **Microbodies** | 1.052 | 0.836 | 0.978 | 0.293 | 0.167 | 0.156 | ANOVA | <0.05 | A AB B |
| **Mitochondria** | 9.363 | 0.861 | 9.086 | 0.732 | 7.005 | 0.143 | ANOVA | <0.001 | A A B |
| **Nucleus** | 10.297 | 1.187 | 12.151 | 0.512 | 5.749 | 2.477 | ANOVA | <0.001 | A A B |
| **Peripheral Bodies** | 0.000 | 0.000 | 1.696 | 0.278 | 0.336 | 0.100 | Mann Whitney U | <0.01 | A B C |
| **Ribosome Cluster** | 20.457 | 2.798 | 0.000 | 0.000 | 0.000 | 0.000 | NA | NA | A B B |
| **Rumposome** | 0.258 | 0.030 | 0.095 | 0.071 | 0.000 | 0.000 | T-Test | <0.001 | A B C |
| **Striated Inclusion** | 0.147 | 0.139 | 0.000 | 0.000 | 0.000 | 0.000 | NA | NA | A B B |
| **Vacuole-bound Lipid** | 0.000 | 0.000 | 0.000 | 0.000 | 3.689 | 1.596 | NA | NA | A A B |
| **Vacuoles excl. Lipid Contents** | 2.322 | 1.453 | 7.560 | 0.852 | 12.958 | 1.780 | ANOVA | <0.001 | A B C |
| **Total Assigned Organelles** | 50.724 | 4.754 | 47.857 | 2.082 | 45.159 | 2.087 | ANOVA | >0.05 | A AB B |
| **Unassigned Cytosol** | 49.276 | 4.754 | 52.143 | 2.082 | 54.841 | 2.087 | ANOVA | <0.05 | A AB B |
| **Vacuoles incl. Lipid Contents** | 2.322 | 1.453 | 7.560 | 0.852 | 16.647 | 0.930 | Kruskal | <0.01 | A A B |
| **Total Lipid Fraction *** | 4.290 | 2.610 | 5.714 | 3.678 | 4.030 | 1.604 | Kruskal | >0.05 | A A A |
| **Total Endomembrane Fraction **** | 4.322 | 1.113 | 11.745 | 1.719 | 20.255 | 1.248 | ANOVA | <0.001 | A B C |

**Supplementary Table 3.** Volumetric percentages and statistical comparisons of cellular structures recorded across chytrid life stages. Data given to 3 decimal places.

****A functional category defined by the sum of cytosolic and vacuole-bound lipids.***

*****A functional category defined by the sum of the endomembrane, Golgi apparatus, microbodies, peripheral bodies, vacuoles incl. lipid contents, and vesicles.***
