# Supplementary material for: A cellular and molecular atlas reveals the basis of chytrid development
Source: eLife. 2022 Mar 1;11:e73933. doi: 10.7554/eLife.73933 (PMC8887899; doi:10.7554/eLife.73933)
Supplement: Supplementary file 4. [file elife-73933-supp4.docx]

**Supplementary Table 4.** Volumetric percentages and statistical comparisons of cell bodies and their corresponding apophyses in immature thalli. Data given to 3 decimal places.

| **Cellular**  **Structure** | **Cellular Structure – Volumetric %** | | | | | |
| --- | --- | --- | --- | --- | --- | --- |
|  | **Cell Body**  **(*n* = 5)** | **±**  **S.D** | **Apophysis**  **(*n* = 5)** | **±**  **S.D** | **Statistical Test used** | ***p*- Value** |
| **Total Volume** | 100.000 | 0.000 | 100.000 | 0.000 | NA | NA |
| **Cell Wall** | 2.409 | 0.328 | 11.034 | 0.534 | Mann Whitney U | <0.01 |
| **Cytosolic Lipid** | 0.341 | 0.159 | 1.308 | 2.318 | Mann Whitney U | >0.05 |
| **Endomembrane** | 2.691 | 0.597 | 12.155 | 5.202 | Mann Whitney U | <0.01 |
| **Glycogen** | 9.399 | 1.969 | 0.000 | 0.000 | NA | NA |
| **Golgi Apparatus** | 0.414 | 0.104 | 1.047 | 0.627 | Mann Whitney U | >0.05 |
| **Microbodies** | 0.167 | 0.156 | 0.000 | 0.000 | NA | NA |
| **Mitochondria** | 7.005 | 0.143 | 6.429 | 4.215 | Mann Whitney U | >0.05 |
| **Nucleus** | 5.749 | 2.477 | 0.000 | 0.000 | NA | NA |
| **Peripheral Bodies** | 0.336 | 0.100 | 0.693 | 1.551 | Mann Whitney U | >0.05 |
| **Vacuole-bound Lipid** | 3.689 | 1.596 | 1.056 | 0.350 | Mann Whitney U | <0.05 |
| **Vacuoles excl. Lipid Contents** | 12.958 | 1.780 | 14.589 | 13.420 | Mann Whitney U | >0.05 |
| **Total Assigned Organelles** | 45.159 | 2.087 | 48.312 | 9.136 | Paired T-Test | >0.05 |
| **Unassigned Cytosol** | 54.841 | 2.087 | 51.688 | 9.136 | Paired T-Test | >0.05 |
| **Vacuoles incl. Lipid Contents** | 16.647 | 0.930 | 15.645 | 13.371 | Mann Whitney U | >0.05 |
| **Total Lipid Fraction*** | 4.030 | 1.604 | 2.364 | 2.407 | Paired T-Test | >0.05 |
| **Total Endomembrane Fraction**** | 20.255 | 1.248 | 29.542 | 9.135 | Mann Whitney U | <0.05 |

****A functional category defined by the sum of cytosolic and vacuole-bound lipids.***

*****A functional category defined by the sum of the endomembrane, Golgi apparatus, microbodies, peripheral bodies, vacuoles incl. lipid contents, and vesicles.***
