# Supplementary material for: A cellular and molecular atlas reveals the basis of chytrid development
Source: eLife. 2022 Mar 1;11:e73933. doi: 10.7554/eLife.73933 (PMC8887899; doi:10.7554/eLife.73933)
Supplement: Supplementary file 5. [file elife-73933-supp5.docx]

**Supplementary Table 5.** Volumetric percentages and statistical comparisons of free-swimming and developing zoospores. Data given to 3 decimal places.

| **Cellular**  **Structure** | **Chytrid Life stage – Volumetric %** | | | | | |
| --- | --- | --- | --- | --- | --- | --- |
|  | **Mature Zoospore**  **(*n* = 5)** | **±**  **S.D** | **Developing Zoospore**  **(*n* = 5)** | **±**  **S.D** | **Statistical Test used** | ***p*- Value** |
| **Total Volume** | 100.000 | 0.00 | 100.000 | 100.000 | NA | NA |
| **Cytosolic Lipid** | 4.290 | 2.610 | 6.766 | 0.859 | Mann Whitney U | >0.05 |
| **Endomembrane** | 0.948 | 0.353 | 1.607 | 0.296 | T-Test | <0.05 |
| **Glycogen** | 1.590 | 1.213 | 5.536 | 1.494 | T-Test | <0.01 |
| **Golgi Apparatus** | 0.000 | 0.000 | 0.367 | 0.156 | NA | NA |
| **Microbodies** | 1.052 | 0.836 | 0.909 | 1.191 | Mann Whitney U | >0.05 |
| **Mitochondria** | 9.363 | 0.861 | 8.400 | 0.403 | T-Test | >0.05 |
| **Nucleus** | 10.297 | 1.187 | 7.202 | 0.361 | T-Test | <0.001 |
| **Ribosome Cluster** | 20.457 | 2.798 | 0.000 | 0.000 | NA | NA |
| **Rumposome** | 0.258 | 0.030 | 0.129 | 0.013 | T-Test | <0.001 |
| **Striated Inclusion** | 0.147 | 0.139 | 0.000 | 0.000 | NA | NA |
| **Vacuoles** | 2.322 | 1.453 | 8.410 | 2.082 | T-Test | <0.001 |
| **Vesicles** | 0.000 | 0.000 | 0.630 | 0.113 | NA | NA |
| **Total Assigned Organelles** | 50.724 | 4.754 | 39.956 | 2.145 | Mann Whitney U | <0.05 |
| **Unassigned Cytosol** | 49.276 | 4.754 | 60.044 | 2.145 | Mann Whitney U | <0.05 |
| **Total Endomembrane Fraction**** | 4.322 | 1.113 | 11.923 | 1.856 | T-Test | <0.001 |

*****A functional category defined by the sum of the endomembrane, Golgi apparatus, microbodies, peripheral bodies, vacuoles incl. lipid contents, and vesicles.***
